# Da-Bu-Yin-Wan and Qian-Zheng-San Ameliorate Mitochondrial Dynamics in the Parkinson’s Disease Cell Model Induced by MPP^+^

**DOI:** 10.3389/fphar.2019.00372

**Published:** 2019-04-24

**Authors:** Cong Gai, Wan-Di Feng, Tian-Yao Qiang, Hao-Jie Ma, Yuan Chai, Shu-Jing Zhang, Zhen-Yu Guo, Jing-Hong Hu, Hong-Mei Sun

**Affiliations:** ^1^Department of Anatomy, School of Preclinical Medicine, Beijing University of Chinese Medicine, Beijing, China; ^2^Center for Scientific Research, School of Preclinical Medicine, Beijing University of Chinese Medicine, Beijing, China

**Keywords:** Parkinson’s disease, SH-SY5Y, MPP^+^, Da-Bu-Yin-Wan, Qian-Zheng-San, mitochondria

## Abstract

To investigate the effect of Da-Bu-Yin-Wan and Qian-Zheng-San (DBYW and QZS) on mitochondrial mass in Parkinson’s disease (PD) cell model induced by 1-Methyl-4-phenylpyridinium Ion (MPP^+^). The SH-SY5Y cell was selected and treated with MPP^+^. The PD model was intervened with DBYW and QZS. CCK-8 method was used to detect the survival rate of cells in each group. Mitochondria was labeled by mitoTracker^®^Red CMXRos probe and observed by laser scanning confocal microscope, and ImageJ software was used to process images and measure mitochondrial form factors; Tetramethylrhodamine methyl ester was used to detect mitochondrial membrane potential (ΔΨm); Luciferase method was used to detect cellular ATP levels; Western-Blot technique was applied to detect the expression levels of Parkin protein, and the expression levels of Mfn1, Mfn2, OPA1, Drp1, and Fis1. We found that DBYW and QZS treatment significantly increased the cell survival rate, form factor (F-factor), mitochondrial activity and ΔΨm after MPP^+^ treatment, while the increase of ATP levels was not significant. In addition, the results of western blot analysis showed that the MPP^+^ induced increase in the expression of Drp1 and Fis1, as well as decrease in Parkin, Mfn1, Mfn2, and OPA1 were all partially revised by DBYW and QZS. In summary, our data strongly suggested that DBYW and QZS treatment can exert protective effects against PD related neuronal injury through regulation the homeostasis between mitochondrial fission and fusion.

## Introduction

Parkinson’s disease (PD) is a progressive degenerative disease of the central nervous system caused by degeneration of dopaminergic neurons in the substantia nigra (Braak et al., 2004). It is mainly characterized by resting tremors, muscle rigidity, slow movement and disorder of postural reflexes, and often accompanied by symptoms of mental disorders, leading to changes in cognitive function, mental state abnormalities (Kehagia, 2016). The etiology of PD is not yet fully understood. It is now widely believed that PD may be caused by the common effects of factors such as heredity, environment, aging, etc. It was found that changes in mitochondrial morphology, dysfunction and unbalanced dynamics were closely related to the pathogenesis of PD (Bose and Beal, 2016; Giannoccaro et al., 2017). Mitochondria are the regulatory centers for cellular metabolism and signal transduction, meanwhile mitochondria are called “power house” of cells. The dynamic characteristics of mitochondria such as mitochondrial fission/fusion, transport and mitochondrial autophagy are important for the maintenance of mitochondrial morphology and function. A large number of studies have shown that mitochondrial dyskinesia is closely related to various neurodegenerative diseases, especially PD.

DBYW and QZS are two TCM formulas that have been applied for more than 600 years. DBYW consists of four Chinese medicinals: *shú dì huáng* (prepared rehmannia root, Radix Rehmanniae Praeparata), *guî bãn* (tortoise plastron, Plastrum Testudinis), *huáng bãi* (amur cork-tree bark, Cortex Phellodendri Chinensis) and *zhî mu* (common anemarrhena rhizome, Rhizoma Anemarrhenae), and it has the effects of enriching yin and supplementing kidney yin and calming the liver and subduing yang. QZS consists of three Chinese medicinals: *jiâng cán* (stiff silkworm, stiff silkorm) and *quán xiç* (scorpion, Scorpio) and *bái fù zi* (typhonium rhizome, Rhizoma Typhonii), and it has the effects of dispelling wind and dissolving phlegm and arresting convulsion. The Chinese medicinals in the two formulas are commonly used for the treatment of PD (Gu and Yuan, 2013; Lu et al., 2016). Our previous research confirmed that DBYW and QZS could regulate brain mitochondrial genes in PD animals and improve mitochondrial morphological structure of tannin neurons (Zhang et al., 2013; Gong et al., 2014). In order to further explore the mechanism of the effects of Chinese herbal compound in preventing and treating PD in mitochondria, the MPP^+^- induced PD cell model was selected to observe the molecular mechanisms of the effects of DBYW and QZS on mitochondria in PD cell model.

## Materials and Methods

### Materials and Reagents

SH-SY5Y cells were purchased from Shanghai Genechem Co., Ltd., DMEM high-glucose medium, Australian-derived fetal bovine serum, trypsin cell digestive juice all from American Invitrogen, MPP^+^ from Sigma Corporation in the US, the required Chinese medicinals from the Qianmen shop of Beijing Tongrentang, CCK-8 kit from Dojindo Molecular Technologies, Inc., MitoTracker^®^Red CMXRos and Tetramethylrhodamine methyl ester (TMRM) from American Life Technologies, ATP detection reagent kit from Beyotime Biotechnology Co., Ltd., BCA protein quantification kit, Parkin Antibody, PINK1 Antibody, TOM20 Antibody, Mitofusin1 Antibody and DRP1 Antibody from American Abcam, Mitofusin 2 Antibody, FIS1 Antibody, Beta Actin Antibody, Alexa Fluor^®^594 Goat Anti-Mouse IgG (H+L), AMCA Donkey Anti-Goat IgG (H+L), FITC Donkey Anti-Rabbit IgG (H+L) and Peroxidas Goat Anti-Mouse IgG (H+L) from American Proteintech Inc., and Peroxidas Goat Anti-Rabbit IgG (H+L) from ZSGB-BIO company.

### Preparation of DBYW Combined With QZS

DBYW (huáng băi 12 g, zhī mŭ 12 g, shú dì huáng 18 g, guī băn 18 g) and QZS (bái fù zi 12 g, jiāng cán 12 g, quán xiē 12 g) were purchased from Tong-Ren-Tang Drugstore in Beijing China, as shown in Table 1. In preparing the decoctions, extract amounts of component herbs were weighed according to the classic percentage and mixed well. The mixture was soaked in distilled water for 30 min and then boiled in eight volumes of water (v/w) for 1 h and extracted twice. After treatment, final concentration of the extract was condensed to 0.5 g/ml. Then the stock solution was filtered through a 0.22 μm membrane pore (Millipore, Billerica, MA, United States), finally divided and stored at -80°C. The marker compounds in DBYW and QZS were analyzed with HPLC-DAD. Chromatograms of the DBYW analyzed with relative reference standards are shown in Supplementary Data Sheet S1.

### Cell Culture and Processing

SH-SY5Y cells were cultured in a DMEM medium containing 10% fetal bovine serum and 1% penicillin under a culture condition of 37°C and 5% CO_2_. A 20 mM MPP^+^ mother liquid was prepared with PBS and stored at -20°C after split packaging. Antibiotic-free medium containing 10% fetal bovine serum was used to prepare a mixed solution containing 1 mM MPP^+^ and 5 μg/ml of the final mixture of Chinese herbal compound, and meanwhile the cells were processed. After 48 h, the cells were collected for indicator detection. The experimental grouping was as followed:

### Cell Survival Rate

Cell survival rate was tested with CCK-8, The medium was incubated for one hour in the incubator and then detected with a microplate reader. The survival rate was calculated as the percentage of absorbance mean value of the experimental group with that of the blank control group, after the cell number in each hole was subtracted from the original number.

### Mitochondrial Morphology

The MitoTracker Red CMXRos (MTR) staining solution was prepared with DMEM to a concentration of 100 nM. Incubated protected from light for 15 min. It was observed with an Olympus FV1000 confocal microscope. Image J (Version 1.48, NIH) image analysis software was used to process the image and obtain data. Mitochondrial morphological parameters, form factors (FF) = perimeter2/(4π × area).

### Detection of Mitochondrial Activity

Specific fluorescence staining of mitochondria of live cell were performed with MTR, the specific method of operation was performed as in 1.5. Images were processed with Image J. and mitochondrial fluorescence intensity (OD value) was calculated.

### Mitochondrial Membrane Potential (ΔΨm)

The mitochondrial probe TMRM was used to mark the mitochondria in living cells to detect mitochondrial membrane potential, it was observed with an Olympus FV1000 confocal microscope. The images were processed with the software Image J, and the mitochondrial fluorescence intensity (OD value) of each image was measured.

**Table 1:** Components of Da-Bu-Yin-Wan and Qian-Zheng-San.

Formula	Chinese name	Pharmacopoeia	Common name	Weight (g)	Voucher numbers
Da-Bu-Yin-Wan	Huang-Bai	Phellodendron chinense Schneid	Amur cork tree bark	60	DBYW01-140406
(DBYW)	Zhi-Mu	Anemarrhena asphodeloides Bunge	Common anemarrhena	60	DBYW02-140406
	Shu-Di-Huang	Schisandra chinensis (Turcz.) Baill	Prepared rehmannia root	90	DBYW03-140406
	Gui-Ban	Paeonia lactiflora Pall	Tortoise plastron	90	DBYW04-140406
Qian-Zheng-San	Bai-Fu-Zi	Rhizoma Typhonii Gigantei	Giant typhonium tuber	60	QZS01-140406
(QZS)	Jiang-Chan	Bombyx batryticatus	Stiff silkworm	60	QZS02-140406
	Quan-Xie	Scorpio	Chinese scorpion (detoxicated)	60	QZS03-140406


### ATP Level

The treated sample was placed in a multi-functional microplate reader to detect with the method of luciferase assay according to the ATP test kit instructions. ATP concentration was calculated in the sample based on the standard curve. The BCA Protein Concentration Assay Kit was used to detect the concentration of protein in the sample, and the ATP concentration was uniformly converted into the protein form of nmol/mg.

### Western Blot Analysis

The cells were lysed in a lysis solution containing a protease inhibitor to extract total cell proteins. The BCA method was used for protein quantification. After the protein was trimmed and denatured, it went through electrophoresis by SDS-PAGE, and was wet-saturated on polyvinylidene fluoride membrane, sealed for one hour at room temperature with 5% skim milk, incubated overnight at 4°C with antibody I, rinsed three times with TBST, incubated with HPR-labeled antibody II for one hour at room temperature, rinsed three times with TBST, and developed for exposure with enhanced chemiluminescence. The image analysis software, Quantity One 4.6.2 was used to analyze images. The relative expression level of the target protein = the value of target band optical density/the value of internal reference protein optical density.

### Statistical Methods

Experimental data were indicated by mean ± standard deviation (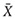 ± s) and the data were processed with one-way analysis of variance (One-way ANOVA) in SAS 8.2. When *P* < 0.05, the difference was statistically significant, and when *P* < 0.01 the difference was more significant.

## Results

### Effect of DBYW and QZS on the Survival Rate of Cells in Each Group

**FIGURE 1:**
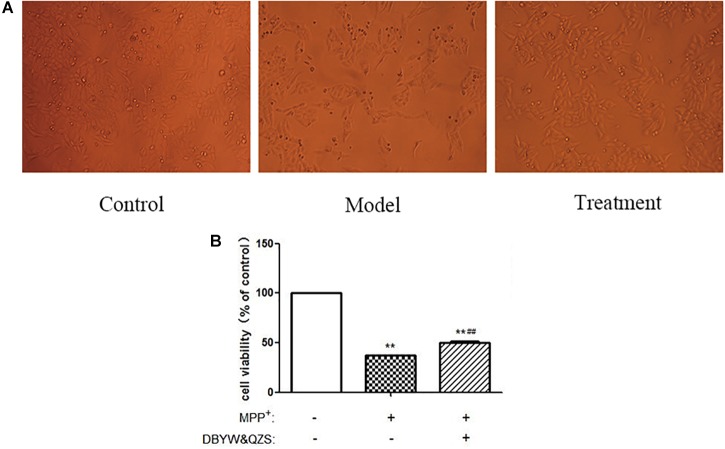
The survival rate and shape changes of SH-SY5Y cells in each group. **(A)** Changes in cellular morphology were observed in cells by inverted phase contrast microscope (10). **(B)**. Cell viability of SH-SY5Y cells were measured after treated with MPP^+^ (1 mM) and DBYW and QZS (5 μg/ml). Data were expressed as mean ± standard deviation (*n* = 3). Compared with the control group, ^∗∗^*P* < 0.01; Compared with the model group, ^##^*P* < 0.01.

The experimental results showed that compared with the normal control group, the cell survival rate after MPP^+^ treatment decreased significantly (*P* < 0.01); compared with the model group, the cell survival rate of the group with the Chinese herbal compound increased significantly (*P* < 0.01). The result was shown in Figure 1.

### Effect of DBYW and QZS on Mitochondrial Morphology in Each Group

**FIGURE 2:**
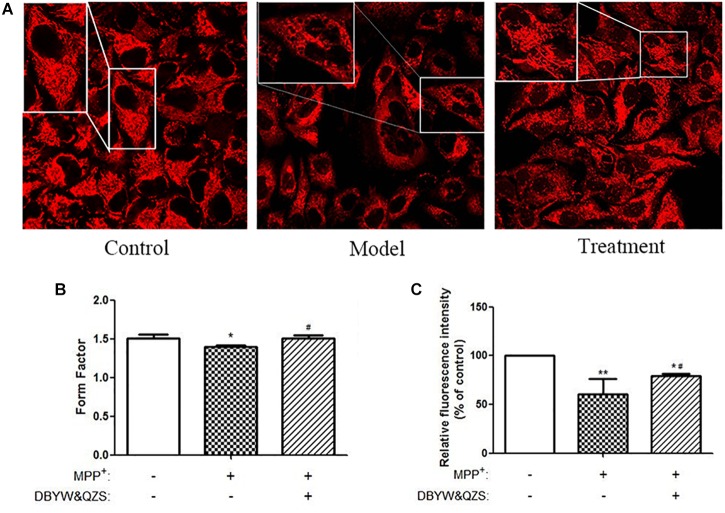
Analysis of mitochondrial morphology and activity. **(A)** Changes in mitochondrial morphology were observed in cells. Mitochondria were labeled by pulsing with MitoTracker Red and assessed by confocal fluorescence microscopy (100). **(B)** FF of mitochondria in each group. **(C)** Analysis of mitochondrial activity. Data were expressed as mean ± standard deviation (*n* = 3). Compared with the control group, ^∗^*P* < 0.05; ^∗∗^*P* < 0.01; Compared with the model group, ^#^*P* < 0.05.

Mitochondria were labeled with the MTR probe, and mitochondrial morphology was observed under a confocal laser scanning microscope, as shown in Figure 2A. The SH-SY5Y cells in the normal control group were found to be plump, with clear nuclei, numerous numbers, and numerous mitochondria, distributed in the perinuclear and protuberances with long lengths. Compared with the normal control group, the number of cells in the model group reduced significantly, vacuole appeared in the cell body, and its mitochondria broke severely, grids decreased, and debris increased. Compared with the model group, the group with the Chinese herbal compound had more large cells, no intracellular vacuoles, and increased number of branches.

Images were uniformly processed using ImageJ software to analyze the morphological parameters of mitochondria: mitochondrial FF, statistical results showed that compared with the normal control group, the mitochondrial FF of the model group reduced significantly (*P* < 0.05), suggesting that the mitochondria rupture with more fragments. Compared with the model group, the mitochondrial FF of the group with the Chinese herbal compound increased significantly (*P* < 0.05), and the mitochondria might have more branches. The results were shown in Figure 2B and the raw data of Figure 2B was shown in Supplementary Table S1.

### Effect of DBYW and QZS on Mitochondrial Activity of Cells in Each Group

In order to detect the effect of the Chinese herbal compound on mitochondrial activity of cells, MTR was used to perform specific fluorescence staining on mitochondria of living cell and they were observed under confocal microscope. The fluorescence intensity was analyzed by ImageJ software. The experimental results showed that compared with the normal control group, the relative fluorescence intensity of mitochondria in the model group (*P* < 0.01) and in the group with the Chinese herbal compound (*P* < 0.05) decreased significantly, suggesting that MPP^+^ reduced mitochondrial activity; compared with the model group, the relative fluorescence intensity of the mitochondria of the group with the Chinese herbal compound increased significantly (*P* < 0.05) and the mitochondrial activity enhanced. The results were shown in Figure 2C.

### Effect of DBYW and QZS on Mitochondrial Membrane Potential (ΔΨm) of Cells in Each Group

**FIGURE 3:**
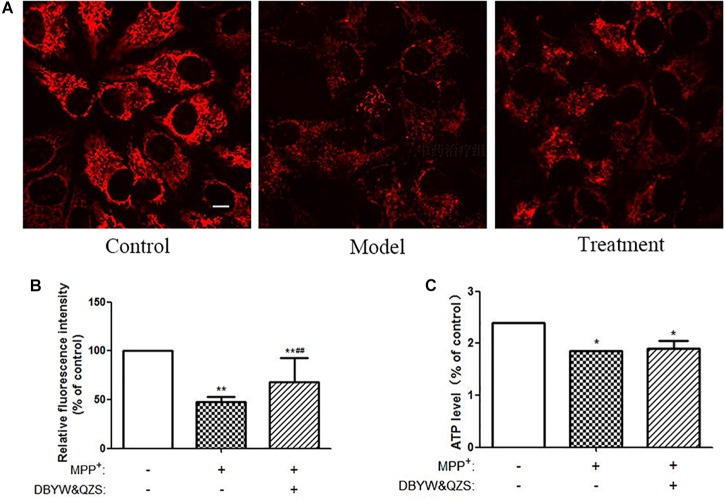
Analysis of mitochondrial function. **(A)** Changes of Mitochondrial Membrane Potential in different groups. TMRM was adopted to label mitochondria of living cells. Adjust LUT to select Jet. The bar was 10 μm. **(B)** Relative fluorescence intensity in each group. **(C)** Relative ATP level of the cells in each group. Data were expressed as mean ± SD (*N* = 3). Compared with the control group, ^∗^*P* < 0.05.

Mitochondrial transmembrane potential is an important indicator of mitochondrial function status. TMRM is a kind of osmotic cationic fluorescent probe. The membrane potential on the functional mitochondrial membrane in living cells is positive externally and negative internally, which can attract the probes into the negative charge on the mitochondrial matrix and intima, thus marking mitochondria. The strengthening or weakness of fluorescence indicates the increase or decrease of mitochondrial inner membrane electronegativity. The results showed that compared with the normal control group, the ΔΨm down-regulated significantly and the relative fluorescence intensity decreased significantly in the model group and in the group with the Chinese herbal compound (*P* < 0.01). Compared with the model group, the relative fluorescence intensity of the group with the Chinese herbal compound increased significantly (*P* < 0.01). The results were shown in Figure 3A,B.

### Effect of DBYW and QZS on ATP Levels of Cells in Each Groups

Compared with the normal control group, the ATP levels in the model group and the group with the Chinese herbal compound decreased significantly (*P* < 0.05), and the model group decreased 22.9% compared with the normal control group, while the group with the Chinese herbal compound decreased 20.8%. The results were shown in Figure 3C.

### Effect of DBYW and QZS on the Expression of Parkin Protein and Mitochondrial Fission and Fusion Proteins of Cells in Each Group

**FIGURE 4:**
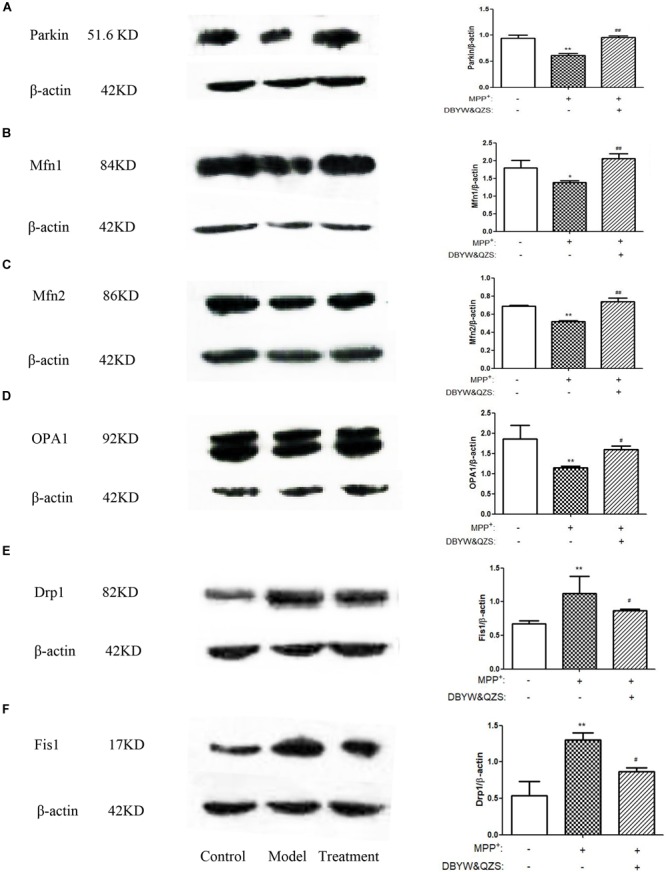
Effect of DBYW and QZS on the expression of Parkin protein and mitochondrial fission and fusion proteins. **(A)** Representative western blots for Parkin and β-actin were shown. And the right bar graph showed the quantification data in arbitrary units (AU) from three independent experiments for the relative level of Parkin, after normalization with respect to β-actin. Experimental groups and treatments were described in Table 2 in the Methodology. Data were expressed as mean ± SD (*N* = 3). Compared with the control group, ^∗^*P* < 0.05; ^∗∗^*P* < 0.01; Compared with the model group, ^##^*P* < 0.01. **(B)** Representative western blots for Mfn1 and β-actin were shown. **(C)** Representative western blots for Mfn2 and β-actin were shown. **(D)** Representative western blots for OPA1 and β-actin were shown. **(E)** Representative western blots for Drp1 and β-actin were shown. **(F)** Representative western blots for Fis1 and β-actin were shown.

The results showed that compared with the control group, the expression of Parkin (*P* < 0.01), Mfn1 (*P* < 0.05), Mfn2 (*P* < 0.01) and OPA1 (*P* < 0.01) in the model group decreased significantly, whereas the expression of the two fission proteins, both Drp1 and Fis1 were increased by MPP^+^ treatment (*P* < 0.01). After treatment with DBYW and QZS, the expression of Parkin (*P* < 0.01), Mfn1 (*P* < 0.01), Mfn2 (*P* < 0.01) and OPA1 (*P* < 0.05) increased significantly, whereas the expression of Drp1 (*P* < 0.01) and Fis1 (*P* < 0.05) were lower than in MPP^+^ treated cells. The results were shown in Figure 4A–F. The original, uncropped image files for the western blots were shown in Supplementary Data Sheet S1.

**Table 2:** Experimental groups and treatments.

Groups	MPP^+^	DBYW and QZS
Control (C)	–	–
Model (M)	1 mM	–
DBYW and QZS (Z)	1 mM	0.5 g/ml


## Discussion

In this experiment, the selected SH-SY5Y cell strain of human neuroblastoma is characterized by its cell morphology, physiology and biochemical characteristics similar to normal neurons, rapid reproduction, and many properties of dopaminergic neurons. Therefore, the cell strain is widely used *in vitro* studies of neurological diseases, especially in PD (Feng et al., 2006; Xie et al., 2010).

MPP^+^ is a neurotoxin that has often been used to establish experimental cell model of PD. Studies showed that MPP^+^ inhibited the activity of mitochondrial complex I, interfered with oxidative phosphorylation, and reduced the synthesis of ATP, thus interrupted the normal operation of the mitochondrial respiratory chain, leading to excessive fragmentization of mitochondria, lack of ATP, loss of mitochondrial membrane potential, and eventually cell death (Zeng et al., 2017; Zhang et al., 2017; Zhong et al., 2018). In this experiment, MPP^+^ was selected to construct the PD cell model. Our experimental results were consistent with the former literature reports, suggesting our successful modeling.

In recent decades, DBYW and QZS were clinically employed to treat PD. Clinical statistics found that the treatment of PD with traditional Chinese formulas in 189 articles, a total of 110 kinds of Chinese medicinals were involved, among which the Chinese medicinals for nourishing the liver and the kidney: *shú dì huáng* was used for 51 times accounting for 2.6%, *guī băn* 38 times accounting for 1.9%, and *zhî mu* 18 times accounting for 0.9%, the three Chinese medicinals are all the components of DBYW; and also among which the Chinese medicinals for dissolving phlegm and extinguishing wind: *jiāng cán* was used for 31 times accounting for 1.5%, *quán xiĕ* 27 times accounting for 1.4%, the two Chinese medicinals are the components of QZS. All the above information provides some basis for the selection of Chinese formulas in our research (Gu and Yuan, 2013). The previous study of our research group also confirmed that DBYW and QZS could reduce MPTP-induced damage to the nucleus membranes and mitochondria in the substantia nigra of PD mice (Zhang et al., 2013; Gong et al., 2014), and we further discovered at the cellular level that DBYW and QZS could increase the length of mitochondria and reduce mitochondrial fragments in SH-SY5Y cells processed with MPP^+^ (Ma et al., 2015). In addition, modern studies found that some active ingredients in DBYW and QZS could protect the activity of substantia nigra DA neurons. Catalpol, a component of *shú dì huáng*, is used in mice with PD and it is found that it can exert a protective effect on DA neurons and improve the behavioral scores of Parkinson’s mice, by up-regulating the content of DA and its major metabolites (DOPAC and HVA) in the striatum and by increasing the number of Tyrosine hydroxylase positive cells and the content of glial cell-derived neurotrophic factor (Xu et al., 2010). Chimonin, one of the important chemical constituents of *Zhī mŭ*, can reduce the loss of dopamine neurons and exert a neuroprotective effect by reducing the content of GSH, MDA, and SOD (Feng et al., 2014). Some active ingredients in *guī băn* can promote the differentiation of neural stem cells into DA energy neurons and save the structure and function of denatured DA energy neurons to protect the central nervous system by increasing the content of bone morphogenetic protein-4 (Xue and Tu, 2017). Scorption venom toxins in *quán xiĕ* have a protective effect on the mitochondrial structure and function of rat brain neurons. SVc inhibits the activity of mitochondrial cytochrome oxidase and succinate dehydrogenase, reduces respiratory control rate, oxidative phosphorylation efficiency and oxygen consumption rate, and increases mitochondrial membrane fluidity. Long-term action of the scorpion venom toxin polypeptide SVIII on mitochondria has an effect on delaying mitochondrial aging (Xing, 2009; Yin et al., 2014). The results of this study are consistent with the previous literature reports. The results showed that DBYW and QZS had a certain protective effect on MPP^+^-induced PD cell model, which could significantly increase cell survival rate and improve mitochondria morphology and activity. DBYW and QZS could increase the mitochondrial membrane potential, but the ATP level in the treatment group did not increase significantly.

**FIGURE 5:**
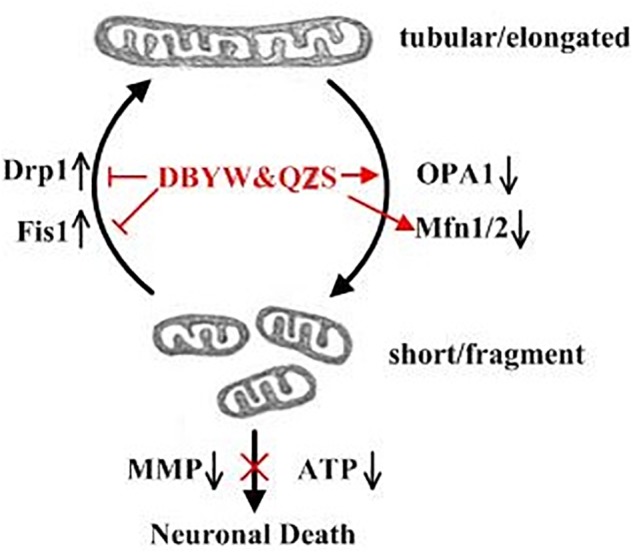
Summary of the DBYW and QZS on mitochondrial dynamics in the PD cell model induced by MPP^+^. Both fission and fusion enable dynamic morphological changes of the mitochondrial network contributing to mitochondrial maintenance, function and quality control. While Mfn1/2 and OPA1 mediate the fusion of the mitochondrial outer and inner membrane, respectively, Drp1 is a key factor in mitochondrial fission. Fis1 localizes on the mitochondrial outer membrane and can recruit Drp1 to participate in the fission of mitochondria. We found that individual mitochondria disclosed a fragmented structure, lack of ATP, loss of mitochondrial membrane potential were more prominent in MPP^+^ treated SH-SY5Y cells. In addition, the MPP^+^ induced increase in Drp1 and Fis1, as well as decrease in Mfn1, Mfn2 and OPA1 were all partially prevented by DBYW and QZS, indicating that DBYW and QZS induced protection against MPP^+^ induced toxicity is mediated by preservation of the balance between mitochondrial fission and fusion.

What is the specific mechanism or effect of DBYW and QZS for protecting mitochondria in PD cell model? One of the most important means that might adapt mitochondrial function to various conditions of living cells is dynamic structural changes of the mitochondrial network, including continuous remodeling by fusion and fission events (Szabadkai et al., 2004). A Study found that the Parkin played an important role in maintaining mitochondrial morphology and function. The Parkin gene (also known as PARK2 gene) encodes an E3 ligase protein Parkin with 465 amino acids. Its N-terminus is a ubiquitin-like domain, and the C-terminus is composed of RING-finger motifs (RING1 and RING2) and in-between RING (IBR) constituting the RING1–IBR–RING2 structure (Wang et al., 2011). Parkin regulated mitochondrial morphology and its remodeling, mainly through regulating the expression of related fission and fusion proteins to maintain mitochondrial homeostasis (Lim et al., 2012; Truban et al., 2017; Chu, 2018). These dynamic processes are of particular importance in neuronal cells because of their post-mitotic state and long processes with higher energy requirements, and dis-regulation in both fission and fusion proteins have been associated with central nervous system diseases (Van Laar and Berman, 2009; Liu et al., 2015). The proteins that regulate mitochondrial fusion mainly include Mfn1 and Mfn2, both of which synergistically promote the fusion process of mitochondrial outer membrane, and optic atrophy protein 1 (Opa1) participates in the fusion of mitochondrial inner membrane. The proteins that regulate mitochondrial fission mainly include Drp1 and Fis1. Fis1 localizes on the mitochondrial outer membrane and can recruit Drp1 to participate in the fission of mitochondria (Itoh et al., 2013). The fusion is crucial for mitochondrial interactions and communication and fission facilitates the segregation of mitochondrial renewal and distribution along the cytoskeletal tracks. Defects in either fusion or fission, leading to mitochondrial fragmentation, decrease the energy production and increase the oxidative stress, which promotes cell dysfunction and death (Gao et al., 2017; Xu and Qin, 2017; Zhao et al., 2017; Zhou et al., 2018). A subset of studies have demonstrated that dynamin-related protein 1 (Drp1), a key factor in mitochondrial fission, is promoted in a Drosophila Parkin mutation model (Lutz et al., 2009). Drp1 overexpression may lead to mitochondrial fragmentation and impair the integrity of mitochondria. Recent studies have also indicated that the expression and steady state of mitochondrial fusion promoting factor MFN is negatively associated with the activity of Parkin in Drosophila (Poole et al., 2010; Yu et al., 2011). Inhibition of mitochondrial fragmentation by activation of Mfn1/2 or OPA1 was shown to antagonize apoptosis progression, whereas the pharmacologic inhibitor of Drp1, mdivi-1, inhibited tBID-dependent cytochrome c release from isolated mitochondria (Lackner and Nunnari, 2010). In our study, individual mitochondria disclosed a fragmented structure, lack of ATP, loss of mitochondrial membrane potential were more prominent in MPP^+^ treated SH-SY5Y cells compared with those cells with DBYW and QZS treatment. In addition, the MPP^+^ induced increase in Drp1 and Fis1, as well as decrease in Mfn1, Mfn2 and OPA1 were all partially prevented by DBYW and QZS (Figure 5). These suggest that the dynamic balance between mitochondrial fission and fusion was disrupted in the PD group by the neurotoxin MPP^+^, causing a series of mitochondrial morphological changes and dysfunction. While DBYW and QZS restored the balance significantly, suggesting that the Chinese herbal compound may protect the neurons by maintaining mitochondrial homeostasis and improving MPP^+^- induced mitochondrial damage.

Taken together, our data demonstrate that DBYW and QZS has a therapeutic potential for the treatment of PD and it can be attributed to its regulation of the dynamic properties of mitochondria.

## Author Contributions

CG and W-DF the contributed to the interpretation of results and writing of the manuscript. H-MS contributed to the study design and interpretation of the results. CG, S-JZ, and J-HH instructed experiments. W-DF, T-YQ, H-JM, and YC conducted the experiments. YC and Z-YG reviewed and approved the manuscript.

## Conflict of Interest Statement

The authors declare that the research was conducted in the absence of any commercial or financial relationships that could be construed as a potential conflict of interest.
