# Supplementary material for: Da-Bu-Yin-Wan and Qian-Zheng-San Ameliorate Mitochondrial Dynamics in the Parkinson’s Disease Cell Model Induced by MPP+
Source: Front Pharmacol. 2019 Apr 24;10:372. doi: 10.3389/fphar.2019.00372 (PMC6491701; doi:10.3389/fphar.2019.00372)
Supplement: Supplementary file 2 [file Data_Sheet_1.ZIP › supplementary material/Notes on Supplementary Materials.docx]

**Fig.4.** **Effect of DBYW & QZS on the expression of Parkin protein and mitochondrial fission and fusion proteins.** (A) Representative western blots for Parkin and β-actin were shown. Experimental groups and treatments were recorded as C(Control), M(Model), Z(DBYW & QZS). (B) Representative western blots for Mfn1 and β-actin were shown. (C) Representative western blots for Mfn2 and β-actin were shown. (D) Representative western blots for OPA1 and β-actin were shown. (E) Representative western blots for Drp1 and β-actin were shown. (F)Representative western blots for Fis1 and β-actin were shown.
