# Supplementary figures and images for: Da-Bu-Yin-Wan and Qian-Zheng-San Ameliorate Mitochondrial Dynamics in the Parkinson’s Disease Cell Model Induced by MPP+
Source: Front Pharmacol. 2019 Apr 24;10:372. doi: 10.3389/fphar.2019.00372 (PMC6491701; doi:10.3389/fphar.2019.00372)

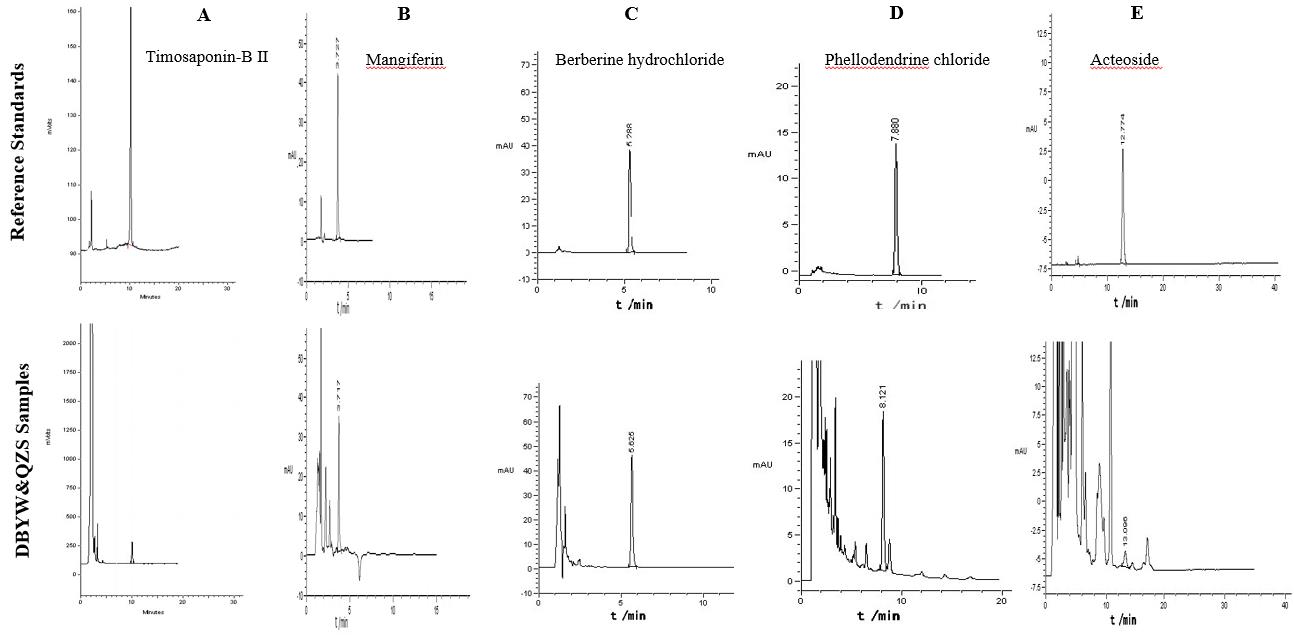

Supplement: Supplementary file 2 [file Data_Sheet_1.ZIP › supplementary material/HPLC-DAD analysis of DBYW&QZS decoction.jpg]

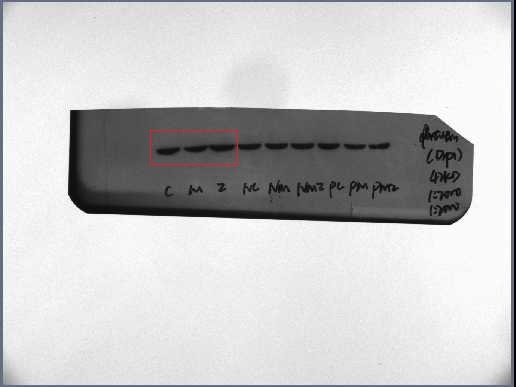

Supplement: Supplementary file 2 [file Data_Sheet_1.ZIP › supplementary material/original, uncropped image files for the western blots/Figure 4 - A/actin.jpg]

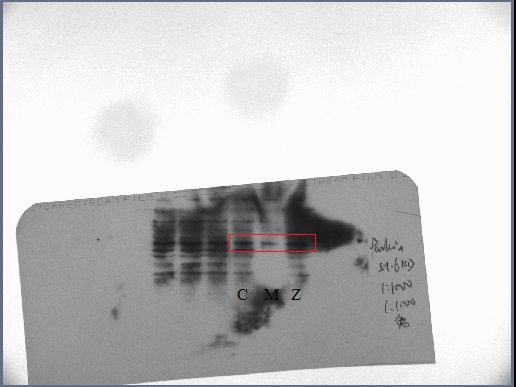

Supplement: Supplementary file 2 [file Data_Sheet_1.ZIP › supplementary material/original, uncropped image files for the western blots/Figure 4 - A/parkin-2 2017-10-24.jpg]

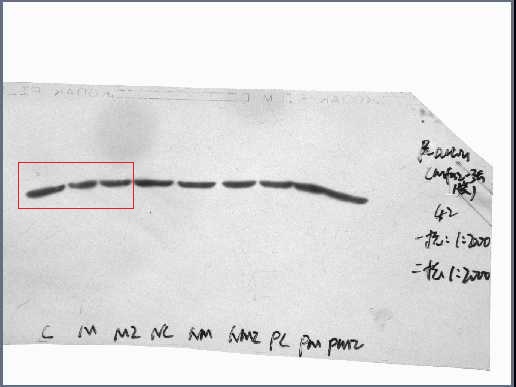

Supplement: Supplementary file 2 [file Data_Sheet_1.ZIP › supplementary material/original, uncropped image files for the western blots/Figure 4 - B/actin.jpg]

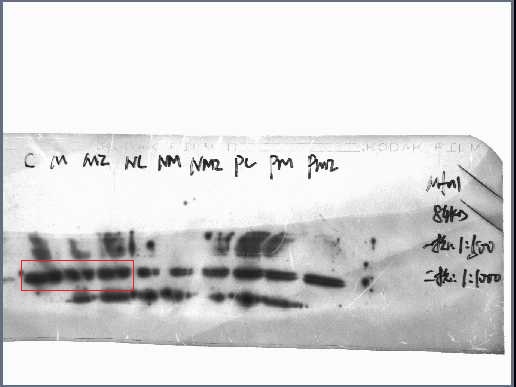

Supplement: Supplementary file 2 [file Data_Sheet_1.ZIP › supplementary material/original, uncropped image files for the western blots/Figure 4 - B/mfn1.jpg]

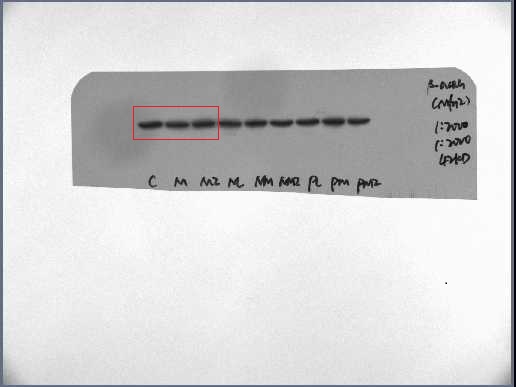

Supplement: Supplementary file 2 [file Data_Sheet_1.ZIP › supplementary material/original, uncropped image files for the western blots/Figure 4 - C/actin.jpg]

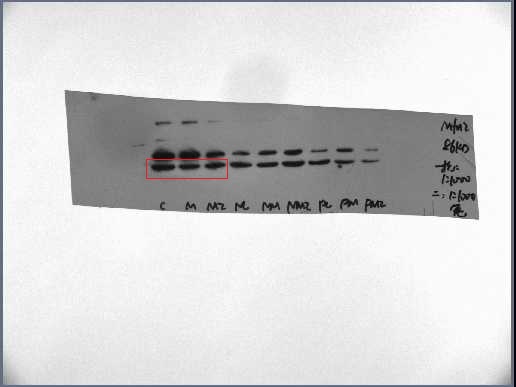

Supplement: Supplementary file 2 [file Data_Sheet_1.ZIP › supplementary material/original, uncropped image files for the western blots/Figure 4 - C/mfn2.jpg]

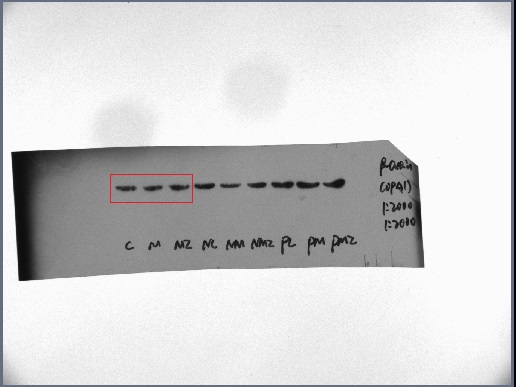

Supplement: Supplementary file 2 [file Data_Sheet_1.ZIP › supplementary material/original, uncropped image files for the western blots/Figure 4 - D/actin.jpg]

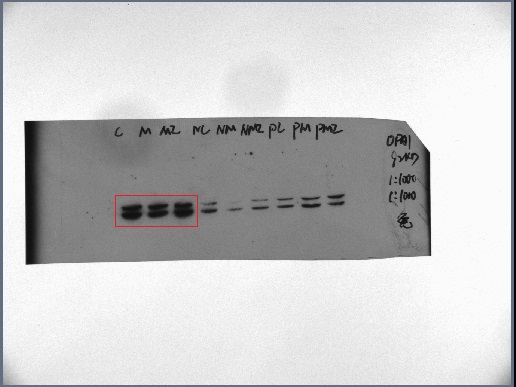

Supplement: Supplementary file 2 [file Data_Sheet_1.ZIP › supplementary material/original, uncropped image files for the western blots/Figure 4 - D/opa1.jpg]

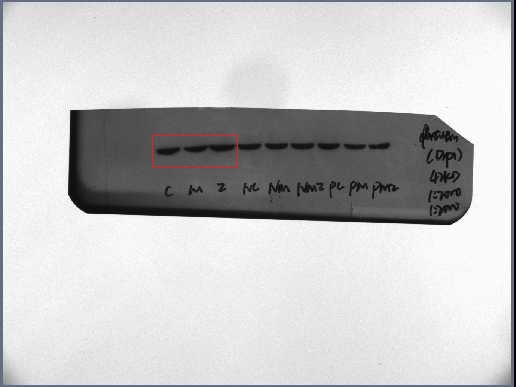

Supplement: Supplementary file 2 [file Data_Sheet_1.ZIP › supplementary material/original, uncropped image files for the western blots/Figure 4 - E/actin.jpg]

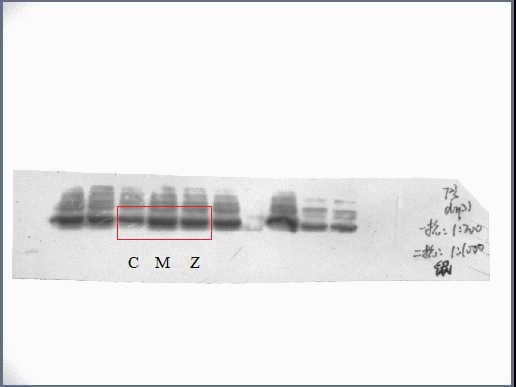

Supplement: Supplementary file 2 [file Data_Sheet_1.ZIP › supplementary material/original, uncropped image files for the western blots/Figure 4 - E/drp1.jpg]

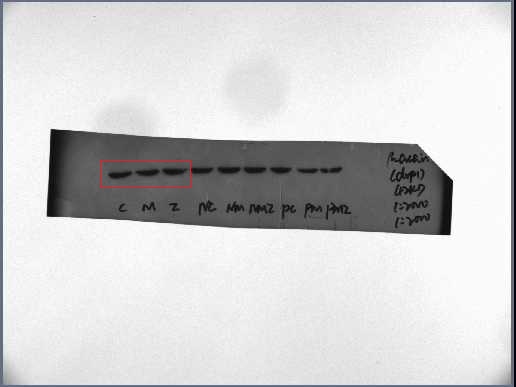

Supplement: Supplementary file 2 [file Data_Sheet_1.ZIP › supplementary material/original, uncropped image files for the western blots/Figure 4 - F/actin.jpg]

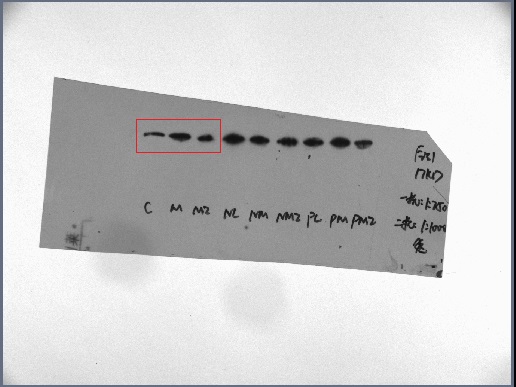

Supplement: Supplementary file 2 [file Data_Sheet_1.ZIP › supplementary material/original, uncropped image files for the western blots/Figure 4 - F/fis1.jpg]
